# The effects of an Aloe-based polyherbal formulation on fertility parameters in male rats

**DOI:** 10.22038/ajp.2024.25197

**Published:** 2025

**Authors:** Fahimeh Safaeinejad, Mojgan Tansaz, Farkhonde Sarhaddi, Vahid Esmaeili, Fatemeh Jafari, Akram Shahhosseini, Behnaz Keramatian, Homa Hajimehdipoor

**Affiliations:** 1 *Traditional Medicine and Materia Medica Research Center, Shahid Beheshti University of Medical Sciences, Tehran, Iran*; 2 *Traditional Medicine and Materia Medica Research Center and Department of Traditional Medicine, School of Traditional Medicine, Shahid Beheshti University of Medical Sciences, Tehran, Iran*; 3 *Department of Pathology, School of Medicine, Zahedan University of Medical Sciences, Zahedan, Iran*; 4 *Faculty of Medical Sciences, Zahedan Branch, Islamic Azad University, Zahedan, Iran*; 5 *Department of Embryology, Reproductive Biomedicine Research Center, Royan Institute for Reproductive Biomedicine, ACECR, Tehran, Iran*; 6 *Traditional Medicine and Materia Medica Research Center and Department of Traditional Pharmacy, School of Traditional Medicine, Shahid Beheshti University of Medical * *Sciences, Tehran, Iran*

**Keywords:** Aloe, Ayarij-e-Faiqra, Male fertility, Busulfan, Iranian traditional medicine

## Abstract

**Objective:**

Aloe dry juice as a purgative agent is widely used in phytotherapy. In Iranian traditional medicine to decrease Aloe side effects, some plants are added, and this polyherbal formulation is named "*Ayarij-e-Faiqra*" (AF). Based on the anti-fertility properties of Aloe, this study investigates the anti-fertility effects of Aloe-based polyherbal formulation to find the impact of accompanying plants on the anti-fertility effects of Aloe.

**Materials and Methods:**

In this study, forty male rats were classified into the following groups: the control group, the sham group receiving only busulfan carrier solution (DMSO 50%) on days 1 and 21 via intraperitoneal injection, the busulfan group received intraperitoneally 10 mg/kg of busulfan on days 1 and 21, the Aloe group received 25 mg/kg of Aloe-dry juice, and the AF group was administered with 71 mg/kg (containing 25 mg/kg of Aloe dry juice). Treatment was performed by gavage for 56 days. Testis weight and histological alterations, sexual hormone levels (testosterone, estrogen, and progesterone), and classical and functional sperm parameters were examined.

**Results:**

Our findings showed that AF negatively affects testicular tissue architecture and sperm quality such as count, motility, morphology, and viability which were accompanied by an imbalance of testosterone, estrogen, and progesterone hormones. In addition, reaction oxygen species (ROS) and apoptosis increased in the sperm cells of the AF group while decreasing their mitochondrial membrane potential.

**Conclusion:**

The plants presented in the formulation of AF cannot cover the anti-fertility side effects of Aloe.

## Introduction

Infertility affects 10-15% of couples, and it is characterized as the inability of couples to have a child after one year despite the non-use of contraceptives and regular unprotected sexual intercourse. Male infertility accounts for about 20-30% of total infertility cases (Babakhanzadeh et al. 2020). The three factors responsible for male infertility include reduced sperm count, weak motility, and abnormal sperm morphology (Babakhanzadeh et al. 2020; Sharma 2017). Other reasons for male infertility include hormonal disorders, physical or lifestyle problems, psychological matters, chromosomal abnormalities, or gene defects. Although many research efforts have recognized the fundamental bases of male infertility, about 70% of cases remain unidentified (Babakhanzadeh et al. 2020).

Among the environmental and nutritional factors, many medicinal plants have anti-fertility properties. Modern scientific research has confirmed the anti-fertility effects of some of these plants (Jain 2015; Kachroo and Agrawal 2011). Various medicinal plant extracts with anti-fertility activity in both male and female sexes have been investigated. Regarding male infertility, some plants have spermicidal properties, while others decrease sperm count, alter sperm motility, and enhance reactive oxygen species (ROS) production. In addition, some plants cause changes in testicular hormones (Khaki et al. 2009), which ultimately deteriorate the reproductive performance.

 It is essential to investigate the biological activity of plant substances in male reproductive functions and detect natural materials with anti-fertility possibilities. Despite the incomplete evidence about the adverse effect of many of these medicinal plants on male fertility, and the available animal data, it is important to be cautious when consuming medicinal plants. Therefore, it is recommended to conduct more research in this regard (Roozbeh et al. 2016).

One of these plants is *Aloe *spp. which has many uses in society, particularly in cosmetics and treatment. There are over 250 species of this plant in the world. The gel-like parts inside the leaves of *Aloe* are both transparent and edible. *Aloe*-dry juice is a strong laxative and contains anthraquinone compounds. Studies have demonstrated that high doses of *Aloe *can reduce the activity of the central nervous system, decrease the number of red blood cells, and seriously damage the morphology of sperm cells (Darabi and Torabzadeh 2016). 

*"Ayarij-e-Faiqra" *(AF) is one of the well-known products of *Aloe* in Iranian traditional medicine (ITM) which exhibits laxative properties (Moein et al. 2017; Razmgah et al. 2021) and is used along with other drugs for constipation and related disorders (Choopani et al. 2017). It is assumed that other botanicals present in AF preparation reduce *Aloe's* adverse effects, thereby enhancing the formula's efficacy as a potent laxative with negligible side effects (Moein et al. 2017; Zargaran et al. 2013). This product is used as a tablet in some Iranian traditional clinics.

Considering that AF is extensively used in the field of traditional medicine, it is crucial to verify its adverse reactions. Therefore, in an oligospermia model using busulfan (Choopani et al. 2017; Zargaran et al. 2013), we compared the influence of *Aloe* alone with AF on fertility parameters to find the effects of other plants of the AF product on male fertility.

## Materials and Methods

### Materials of plant

Plants used in the experiment (Table 1) were purchased from an herbal market in Tehran and identified in the Herbarium of TMRC. 

### Preparation of Ayarij-e-faiqra tablet and standardization

*Ayarij-e-faiqra* tablets were prepared with a mixture of *Aloe* juice (250 mg), *Pistacia atlantica* oleogumresin (57.5 mg), and *Piper cubeba*,* Nardostachys jatamansi*, *Cinnamomum verum*, and *Cinnamomum cassia*, *Rosa* sp. (38.5 mg each one) along with 210 mg excipients. Tablets were pressed using a single-punch tablet manufacturing apparatus. To standardize *Aloe* juice and the tablet, hydroxyanthracene derivatives, expressed as barbaloin were determined in the samples (Cartwright 2016).

### Pharmacological study

Forty Wistar rats, aged 8-10 weeks and with weights ranging between 180- 200 g, were allocated into five groups, each including eight rats. Grouping was as follows: control group, the sham group that received only busulfan carrier solution dimethyl sulfoxide 50% (DMSO) on days 1 and 21 via intraperitoneal (*i.p*) injection. Other groups included the busulfan (Sigma, St. Louis, MO, USA) group received 10 mg/kg of busulfan *i.p* on days 1 and 21, the *Aloe* group received 25 mg/kg of *Aloe*-dry juice, and the AF group was administered with 71 mg/kg (contains 25 mg/kg of *Aloe* dry juice). 

Administration of the extract was conducted for 56 consecutive days by gavage. This period was chosen based on the spermatogenesis duration in rats (Shokri et al. 2014). The dosages of plants applied in this study were determined based on the dosages recommended by the Iranian traditional medicine.

### Sample collection

After 56 days of treatment, blood samples were acquired via cardiac puncture, and then centrifuged at a rate of 3500 rpm for 15 min. Sera were isolated and used for sex hormones examination. Next, testis and epididymis were gained from the abdominal cavity. The weight of the testis was measured using a digital scale. The samples from epididymis tissue were employed in the investigation of sperm parameters.

### Histological procedure

Testicular tissue samples were immersed in Bouin’s solution for 24 hr for histological assessment. Next, it was washed in tap water followed by dehydration via graded ethanol concentrations. Samples were preserved in a 10% formalin buffer solution and subsequently surrounded in paraffin blocks. Afterward, tissue preparations were done into 5-μm thin sections and followed by staining with hematoxylin and eosin (H&E). Then, the alterations in the seminiferous tubules were examined. The spermatogenesis and the statue of seminiferous tubules were assessed under a light microscope.

### Classical sperm parameters

The tail epididymis was cut into small parts within Ham's F-10 culture medium and incubated at a temperature of 37°C for 30 min. The sperms within the epididymis were quantified using a standard technique of slide hemocytometry. Then, 10 μl of the semen was dropped onto the hemocytometer via a Neubauer chamber (Deep 1/10 mm, LABART, Germany) defined by Pant and Srivastava (Bahrami et al. 2018). Then, at a magnification of ×40, the sperm cells were counted as one million spermatozoa per ml of suspension. Sperm motility (comprising progressive, non-progressive, and immotile categories) was examined via an optical microscope (manufactured by Olympus Optical Co., Japan) in 5 sequential estimations and the average is reported. 

To determine sperm viability and morphology, a 50 μl suspension of sperms was gently mixed with a 50 μl eosin-nigrosin solution including 1.67% eosin, 10% nigrosin, and 0.1 M sodium citrate. Subsequently, 10 μl of this resultant mixture was transported to a glass slide and uniformly spread using another slide. Following the preparation of the smear, sperm viability and morphology were examined. Sperms that displayed a white head were considered viable, while those with a redhead were judged as dead. Additionally, the sperms were separated and categorized as normal or abnormal. The abnormality percentage was calculated for each group (Hamilton et al. 2015).

### Determination of hormones in the serum

Concentrations of Follicle-stimulating hormone (FSH), luteinizing hormone (LH), and testosterone (T) hormones in the blood serum of animals were measured using assay kits through the enzyme-linked immunosorbent (ELISA) method. The ELISA kits included Rat T ELISA Kit (Cat. Number. MBS282195), Rat LH ELISA Kit (Cat. Number. ER1123), and Rat FSH ELISA Kit (Cat. Number. ER0960).

### Intracellular ROS evaluation

The intracellular reactive oxygen species (ROS) level assessment was conducted via the use of Dichloro-dihydro-fluorescein diacetate (DCFH-DA) at the concentration of 25 μM. This compound was separately added to fractions having 1–2×10^6^ sperm/ml and incubated at room temperature for 40 min, in the darkness. Then, each sample group was examined by a flow cytometer containing a 488 nm argon laser (Becton Dickinson FACScan, San Jose, CA, USA). The green fluorescence emitted by DCFH-DA in the range of 500–530 nm was assessed with an excitation wavelength at 488 nm and through the FL-3 channel (Fatemi et al. 2013).

### Flow cytometry assay of apoptosis

Apoptosis of sperm cells was estimated through a flow cytometric method using a commercial PS Detection Kit (IQP, Groningen, and the Netherlands). This specific kit can identify viable, apoptotic-like, and dead sperm cells, based on the manufacturer’s guidelines (Dodaran et al. 2015). To find the percentage of apoptosis in the semen sample, sperm cells were purified in a calcium buffer solution. Next, 10 μl Annexin V-FITC (A) was added to 100 μl semen suspension and incubated for 20 min on ice. Subsequently, 10 μl Propidium Iodide (PI) was added to the suspension and incubated for a minimum of 10 min on ice. Each tube was analyzed by flow cytometry equipment (Becton Dickinson, San Khosoz, CA, USA). The emission of Green fluorescence was identified in FL1 with a band-pass filter of 530/30 nm and red fluorescence (propidium) was observed in FL2 by a band-pass filter of 585/42 nm. The different patterns of labeling in the AN/PI analysis were categorized as follows: viable (A^-^/PI^-^); viable but PS translocated (A^+^/PI^-^); nonviable and PS translocated (A^+^/PI^+^), and nonviable and necrotic sperm (A^-^/PI^+^) (Gholami et al. 2023).

### The evaluation of mitochondrial membrane potential (ΔΨm)

To determine the mitochondrial membrane potential (ΔΨm) of the cells by flow cytometry, sperms were centrifuged at approximately 500 g for 5 min to isolate the extender from the cell pellet. The sperm pellet was submerged in phosphate-buffered saline (PBS) at a pH of 7.4, thus, a solution with around 2×10^6^ sperms per milliliter was achieved. A lipophilic cationic fluorescent dye JC-1 (JC-1, Sigma, St. Louis, MO, USA) was employed for the evaluation of the mitochondrial membrane potential of sperms. In this procedure, 6 μl of JC-1 (153 μM) was added to 2×10^6^ sperm in 200 μl of PBS buffer. After incubation for 15 min at 37°C in darkness, samples were assessed via the flow cytometer using argon-ion laser excitation (488 nm), enabling concurrent readings on FL 1 (530/30 nm) and FL 2 (575/24 nm). JC-1 exhibited a unique capability in labeling and differentiating the high and low membrane potential of mitochondria. While JC-1 in high potential of mitochondria accumulated multimeric and emitted an orange dye (at 590 nm wavelength), in low Δ Ψm, JC-1 existed as a monomer and produced a green dye (ranging between 525 and 535 nm) (Gholami et al. 2023)  

### Statistical analysis

The data analysis was done by GraphPad Prism and flow cytometry Data was analyzed by FlowJo software. Data is presented as mean±SD and differences between the control and treatment groups were examined by the One-way analysis of variance followed by Tukey’s posttest. In all tests, p-value <0.05 was considered significant. 

## Results

### Standardization of the samples

The percentage of hydroxyanthracene derivatives, expressed as barbaloin in *Aloe* juice and AF tablet was found to be 14.13 and 4.31 %, respectively.

### Testis weight

**Figure 1:**
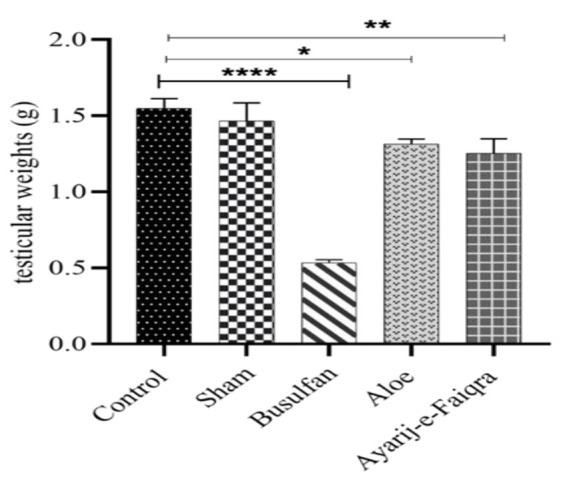
Testicular weight in control, sham, Busulfan, *Aloe,* and AF groups compared to control. (*p<0.5, **p<0.01, and ****p<0.0001)

Data showed a significant alteration in the weight of the testis due to busulfan administration compared to control (p<0.0001). Atrophy of the testis was also observed in the Aloe (p<0.05) and AF (p<0.01) groups in comparison to the control (Figure 1). The sham group had no differences with the control. There were no significant differences between the *Aloe* and AF groups.

### Classical sperm parameters

 Results of epididymis sperm analysis from all groups are detailed in Table 2. The analysis of sperm motility revealed a notable reduction in the total motility of sperms in the Busulfan, *Aloe*, and AF groups compared to control (p<0.05). The sham group had no differences with the control. Moreover, the progressive motility of the busulfan, *Aloe*, and AF had a significant decrease compared to the control group (p<0.05). No significant differences were detected between the *Aloe* and AF groups.

Analyzing sperm count among the 5 groups by a one-way-ANOVA test, displayed that busulfan, *Aloe*, and AF significantly decreased the sperm count of rats as compared to the control group (p<0.0001 for all cases). The sham group had no differences with the control. No significant differences were detected between the *Aloe* and AF groups. 

The viability of sperm markedly declined after exposure to busulfan compared to the control group (p<0.001). The sham group had no differences with the control. Furthermore, viable sperm percentage in the *Aloe*, and AF groups was significantly lower than the control group (p<0.001). There was no significant difference between the *Aloe* and AF groups. 

**Figure 2:**
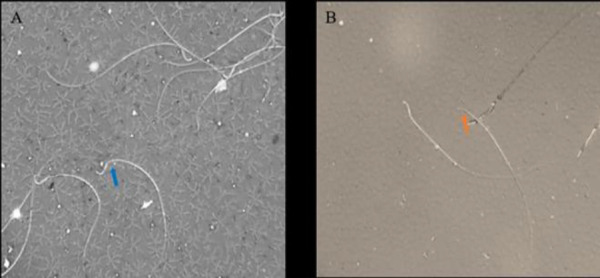
Abnormalities of sperm samples in the busulfan, *Aloe,* and AF groups. A) Bent neck, and B) flat head**.**

The percentages of normal-shaped spermatozoa are shown in Table 2. Morphological analysis of semen samples demonstrated a significantly lower percentage of normal morphology spermatozoa in the busulfan, *Aloe*, and AF experimental groups compared to control *(*p<0.001 for all cases). The sham group had no differences with the control. The common head abnormalities included flatted heads and bent necks observed in sperm samples of these groups (Figure 2).

### Intracellular ROS evaluation

**Figure 3:**
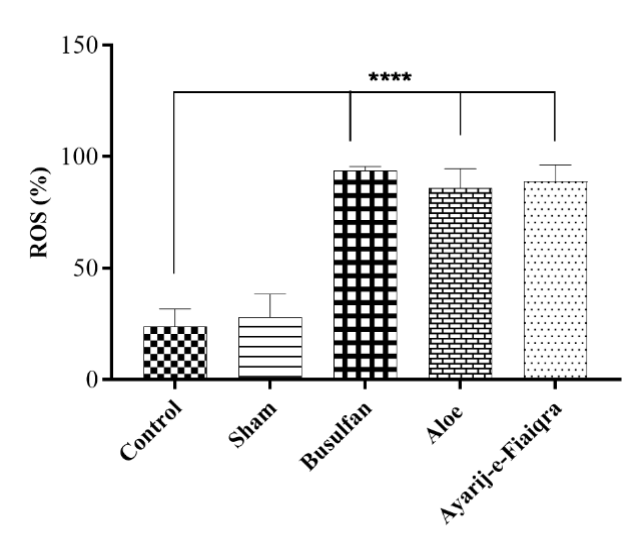
Flow cytometry of ROS percentage in sperm samples of rats (mean±SD) (****p<0.0001 compared to control)

To determine the oxidation effects of *Aloe *and AF, the ROS content of sperms was assessed. Results showed that the sham (28.10±10.18%), Busulfan (93.57±1.95%), *Aloe* (86.03±8.47%)*, *and AF (88.93±7.42%) had a significant difference compared to the control group (23.65±7.99) (p<0.0001 for all cases) and they increased intracellular ROS of sperms. The sham group had no differences with the control. No significant differences were detected between the *Aloe* and AF groups. The percentage of ROS in the different groups is shown in Figure 3.

### Apoptosis assay

**Figure 4:**
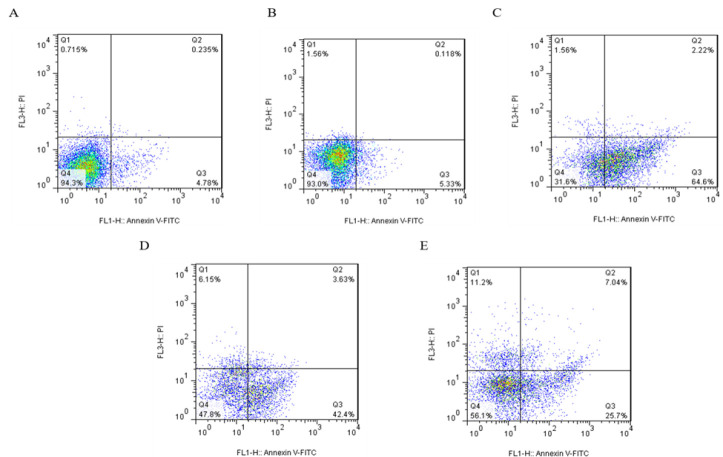
The flowcytometry results of apoptosis in different groups: A) control B) sham C) busulfan D) Aloe E) Ayarij-e-Faiqra. Q1: Necrosis (A^-^/PI^+^), Q2: Late Apoptosis or nonviable and PS translocated (A^+^/PI^+^), Q3: Early Apoptosis or viable but PS translocated (A^+^/PI^-^), Q4: Live Cells or viable (A^-^/PI^-^).

The basic characteristic of apoptosis is the disorganization and lack of coherence of the plasma membrane. In the initial phase of apoptosis, phosphatidylserine (PS) molecules relocate from the membrane to the extracellular situation, which was identified by Annexin V, in the existence of Ca^2+ ^ions. For diagnosis of apoptotic necrotic procedures, propidium iodide (PI) was employed to specify the necrosis of the cell. The sham group had no differences with the control. The results revealed that busulfan meaningfully increased the apoptotic cells. *Aloe* and AF groups also had increased percentages of apoptotic sperms compared to control (Table 3 and Figure 4). There was no significant difference between the *Aloe* and AF groups.

### The evaluation of mitochondrial membrane potential (ΔΨm)

**Figure 5:**
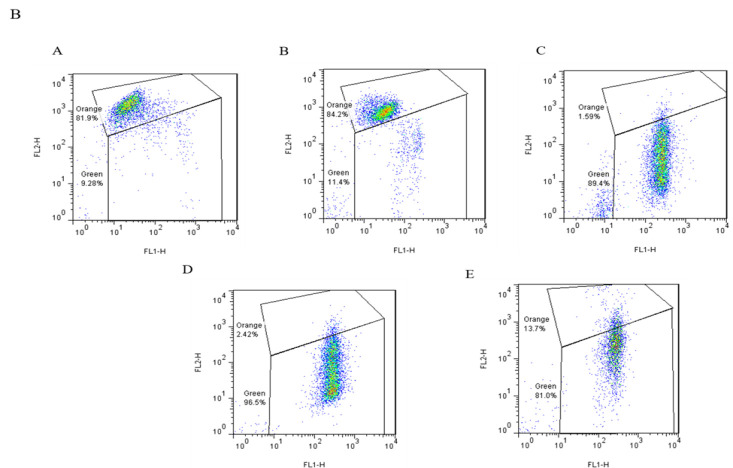
A) The mitochondrial membrane potential (staining orange) of Sperms (mean±SD) (****p<0.0001 compared to control). B) The flowcytometry results of mitochondrial membrane potential in different groups: A) control B) sham C) busulfan D) *Aloe* and E) *Ayarij-e-Faiqra.*

Flow cytometry analysis showed that the percentage of spermatozoa staining orange in the busulfan, *Aloe,* and AF groups (2.00±0.59, 4.03±1.74, 12.55±1.63%, respectively) was significantly lower than the control group (83.05±1.63%) (p<0.0001 for all cases) (Figure 5). The sham group (68.05±8.83%) had no differences with the control. There were no significant differences between the *Aloe* and AF groups. 

### Histological evaluation of testes

**Figure 6:**
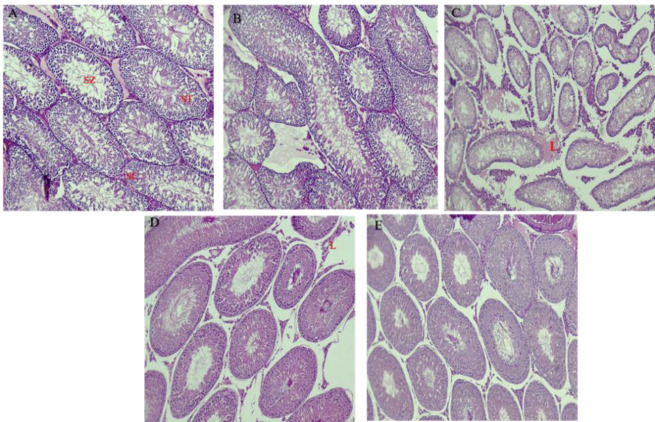
Histopathologic data using hematoxylin-eosin staining, A) control group B) sham C) Busulfan D) *Aloe* and E)* Ayarij-e-Faiqra*. L: Leydig cell, SC: Primary spermatocytes, ST: Spermatids, Sz: sperms.

Histologic sections showed seminiferous tubules composed of Sertoli cells (large cells with an oval nucleus), primary spermatocytes (small cells with a dark nucleus on the basement membrane), spermatids (center of the lumen), and sperms. These findings show benign testis with spermatogenesis. Contrary to that, in the busulfan group, seminiferous tubules were composed of Sertoli cells and primary spermatocytes with the halt of the maturation sequence in them (>90%). In addition, the number of seminiferous tubules decreased in the busulfan group. Few/ no spermatids or spermatozoa were present. The histology of *Aloe* and AF groups had no differences compared to the control group but a reduction in the seminiferous tubules was observed (Figure 6). The sham group had no differences with the control.

### Serum hormone levels

**Figure 7:**
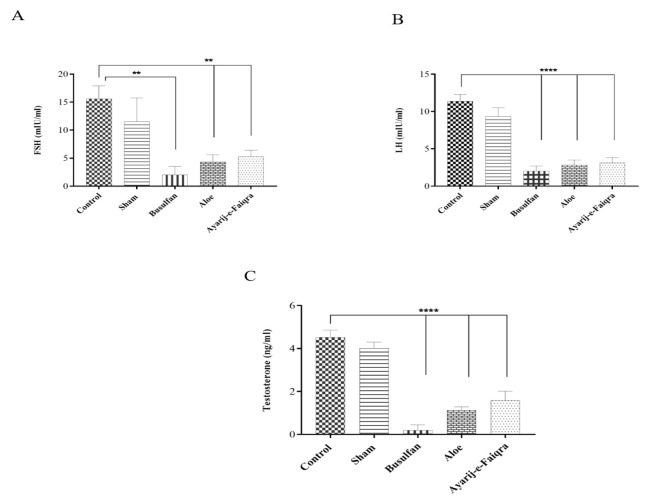
Diagrams of hormone levels in the studied groups: A) FSH, B) LH, and C) Testosterone (**p<0.01, ***p<0.001, and ****p<0.0001 compared to control).

Busulfan caused a decrease in FSH (2.00±1.51 mIU/ml), LH (2.02±0.68 mIU/ml), and T (0.2±0.25 ng/ml) levels compared to the control (15.59±2.33 mIU/ml, 11.38±0.9 mIU/ml, 4.52±0.33 ng/ml, respectively) (p<0.0001 for all cases). *Aloe* and AF also significantly decreased sex hormones levels in comparison to the control group (Figure 7). The sham group had no differences with the control. The level of FSH, LH, and Testosterone in the *Aloe *group was 4.31±1.32 mIU/ml, 2.82±0.68 mIU/ml, and 1.13±0.15 ng/ml, respectively, for the AF group FSH, LH, and Testosterone concentrations were respectively 5.31±1.11 mIU/ml, 3.13±0.68 mIU/ml, 1.59±0.43 ng/ml. There were no significant differences between the *Aloe* and AF groups. 

## Discussion

*Aloe*, with usage in cosmetic, nutrition, and medical domains, has become extremely popular in recent years. In this research, *Aloe*-based polyherbal formulation administration in rats caused a significant decrease in testicular weight, and count, and motility of epididymal sperm, as well as sperm abnormality, the same as the *Aloe* group. Anthraquinones are the main constituent in *Aloe* that act as a laxative agent and potentially cause hypoglycemia (Babu and Noor 2020; Hamilton et al. 2015; Vinson et al. 2005). Consequently, this can lead to metabolic alterations resulting in diminished sperm count and motility (Nowicka-Bauer and Nixon 2020; Vavaiya et al. 2007).

The reduction in testicular weight may be due to the reduction in sperm cell generation from the testis caused by a diminution in the number of seminiferous tubules which account for approximately 80% of the volume of the testes (Abdel-Razik et al. 2021; Oyewepo et al. 2011). Previous studies also demonstrated that there was a decline in testicular weight, sperm count and motility, and spermatozoa abnormalities with *Aloe *(Jiwantare and Dhurvey 2022; Oyewopo et al. 2011; Suardita et al. 2013). In comparison with the results of Nwanjo and Oze who documented that *Aloe* had many antioxidant substances which diminish lipid peroxidation and scavenge free radicals (Nwanjo and Oze 2006).

Asgharzade *et al.* displayed the ability of *Aloe* to lessen testicular weight, levels of serum testosterone, and sperm count in male rats (Asgharzade et al. 2015). This finding is consistent with Oyeyemi and Ajani who reported increased sperm structural abnormalities (Oyeyemi and Ajani 2015).

Farnsworth and Waller found that spermicide properties of plants are related to several structural types of saponins that have astringent activities on the cell membrane of sperm cells, destroying the cell membrane and subsequently, reducing sperm motility, and inhibiting specific enzymes essential for spermatogenesis (Farnsworth and Waller 1982; Paul et al. 2010). Therefore, single or multiple phytochemical constituents could be involved in the significant reduction of sperm number and motility (Wendmu et al. 2020). 

Aloe-emodin and coumaric acid present in *Aloe* are effective in the production and secretion of FSH hormone from the pituitary gland and testosterone from the testes. The administration of AF in the present investigation resulted in a significant reduction in the FSH and LH concentrations. These findings are consistent with previous research by Shariati and Mokhtari who revealed that the *Aloe* administration in rats led to a significant decline in FSH and LH levels (Shariati and Mokhtari 2009). The bioactive compounds within *Aloe* extract, containing aloe-emodin, have been demonstrated to directly impact gonadotropin receptors or the pituitary gland and can alter the concentrations of LH and FSH (Dodge 1998; Jiwantare and Dhurvey 2022).

In addition, the serum concentration of testosterone in the AF group was decreased. Karimi et al. also revealed a significant decrease in testosterone concentration after *Aloe* administration (Karimi Jashni H et al. 2022). Compounds present in *Aloe*, like coumaric acids, stimulate the testicular macrophage actions to produce nitrous oxide (Chrousos 2004). Studies have indicated that paracrine nitrous oxide considerably affects Leydig cell steroidogenesis by inhibiting the heme-containing steroidogenic enzyme CYP17A1, which resulted in the suppression of cholesterol conversion into pregnenolone. Therefore, testosterone production is inhibited (nee Pathak and Lal 2009).

Sperm morphological changes at the head and tail of sperms were observed in the *Aloe*-based formulation group of the rats. This result is consistent with Oyeyemi and Ajani who reported increased sperm structural abnormalities. Androgens are vital for the survival and physiological maturing of sperms in the epididymis (Wendmu et al. 2020). The detected abnormalities of sperm cell morphology could be due to a lack of androgen consequent to the anti-androgenic properties of AF (Oyeyemi and Ajani 2015).

According to the search results, there is evidence that *Aloe* can produce ROS. The fact that oxidative stress plays a main role in cell apoptosis is broadly accepted (Maqbool et al. 2020). The flow cytometry analysis revealed that AF treatment resulted in an increase in ROS production and apoptosis in spermatozoa, in comparison to the control group.  Many studies also approved that plant extracts tempt ROS production leading to cell death (Ramalingam and Rajaram 2018; Vallejo et al. 2017). Aloe-emodin, isolated from *A. vera* has been revealed to ROS production (Chiu et al. 2009; Farshori et al. 2022). Additionally, ROS is involved in mitochondria depolarization and the progression of apoptosis (Redza-Dutordoir and Averill-Bates 2016). The deficiency of mitochondrial membrane potential is a primary step of the apoptosis process (Farshori et al. 2022). Our results confirmed that *Aloe* and AF groups caused a significant decline in mitochondrial membrane potential (ΔΨm). Other studies showed that the extract of plants could induce cytotoxicity over mitochondrial depolarization (Redza-Dutordoir and Averill-Bates 2016).

Finally, our data propose that although the other botanicals existing in AF formulation reduce the digestive effects, but cannot decline *Aloe* side effects on fertility in male rats.

Data exhibited that consumption of the AF significantly increases the risk of pathological tissue damage in testes and decreases reproductive performance in rats. In addition, it was observed that AF led to increased levels of intracellular oxidative stress as well as structural damage to mitochondria and cell apoptosis. Therefore, accompanying other species with *Aloe* and AF has no effects on the anti-fertility side effects of *Aloe* alone.

**Table 1:** Components of *Ayarij-e-Faiqra*

No.	Scientific name	Family	Common name	Part used	code
**1**	*Aloe* spp.	Liliaceae	Bitter aloe	Dry juice	HMS-579
**2**	*Cinnamomum verum*	Lauraceae	Cinnamon	Bark	HMS-575
**3**	*Cinnamomum cassia*	Lauraceae	Cassia, Chinese cinnamon	Bark	HMS-577
**4**	*Nardostachys jatamansi*	Valerianaceae	Indian valerian, spikenard	Rhizome	HMS-574
**5**	*Pistacia atlantica*	Anacardiaceae	Saghez	Oleogumresin	HMS-573
**6**	*Piper cubeba*	Piperaceae	Cubebs	Fruit	HMS-576
**7**	*Rosa* sp.	Rosaceae	rose	Flower	HMS-578

**Table 2:** Parameters of rat sperm quality including count, total motility, progressive motility, morphology, and viability (mean±SD) (n=8 in each group) (*p<0.05, ***p<0.001, and ****p<0.0001 compared to control)

**Parameters**	**Control**	**Sham**	**Busulfan**	***Aloe***	***Ayarij-e-Faiqra***
Count (×10^6^ sperm/ml)	83.38±42.56	70.75±36.38	38.67±23.05^****^	40.87±14.44^****^	39.58±18.22^****^
Total Motility (% motile sperm)	69.00±17.11	60.60±14.39	26.75±11.34^****^	31.25±5.25^****^	32.95±11.4^****^
Progressive motility (%)	28.99±7.4	26.88±8.3	6.79±4.8^*^	7.33±6.85^*^	8.43±6.15^*^
Morphology (% normal sperms)	93.90±5.4	88.27±11.5	32.40±9.2^***^	38.6±12.3^***^	41.20±10.7^***^
Viability (% viable sperm)	89.20±10.5	78.6±14.9	36.50±4.2^***^	41.6±3.51^***^	43.23±5.41^***^

**Table 3:** Apoptosis in sperm samples of rats (mean±SD) (**p<0.01 and ***p<0.001 compared to control)

**Parameters (%)**	**Control**	**Sham**	**Busulfan**	***Aloe***	***Ayarij-e-Faiqra***
Live cells	97.27±2.56	93.5±1.22	25±8.63^****^	51.95±5.87^***^	53.35±10.11^***^
Early apoptosis	1.452±2.23	4.245±2.09	66.5±2.69^****^	34.05±11.81^**^	26.35±8.84^**^
Late apoptosis	0.086±0.08	0.727±0.43	2.4±0.26^*^	5.31±2.4^**^	6.58±0.84^**^
Necrosis	1.18±1.05	1.539±1.25	5.83±6.04	8.92±3.92	13.7±2.12^*^
